# Donation after circulatory death in adult congenital heart disease: A fragile match

**DOI:** 10.1016/j.jhlto.2025.100414

**Published:** 2025-11-12

**Authors:** Jose B. Cruz Rodriguez, Deepak Acharya

**Affiliations:** a Division of Cardiovascular Medicine, University of California San Diego, San Diego CA; b Division of Cardiology, University of Arizona Sarver Heart Center, Tucson AZ

The population with adult congenital heart disease (ACHD) continues to grow as survival improves following the childhood medical and surgical care, and adult survivors now outnumber the children with CHD^1^. Despite this progress, it is estimated that heart failure is the leading cause of premature morbidity and mortality in this population, accounting for up to 40% of deaths in ACHD. This is likely multifactorial as a result of palliated circulations, residual cardiac lesions, multiorgan dysfunction and aging^2^. Given the abnormal structural anatomy and prior surgeries, most patients with ACHD are poor candidates for durable mechanical circulatory support^3^. Therefore, there is a growing need for heart transplantations in ACHD.

Heart transplantation in ACHD warrants specific consideration. These patients have typically undergone multiple sternotomies, elevated pulmonary pressures, higher likelihood of sensitization, may have multiorgan dysfunction including important hepatic dysfunction, and have longer, more complicated transplant surgery^4^. The 5-year mortality rate of an average 40-year-old Fontan patient is comparable to that expected in a 75-year-old individual without ACHD^1^. On the other hand, although ACHD transplant recipients have higher early post-transplant mortality, they have superior long-term conditional survival compared with non-ACHD patients^5^.

Heart transplantation continues to be limited by donor organ availability. In an effort to increase the donor pool for transplantation, donation after circulatory death (DCD) use keeps expanding, with growing data suggesting comparable short- and long-term survival and safety compared to donation after brain death (DBD)^6^, ^7^.

Myocardial ischemia is the main obstacle in DCD organ transplantation. The ischemic DCD hearts are either reperfused outside the donor's body (*ex-situ reperfusion*) on devices or reperfused within the donor's body by in-situ reperfusion using normothermic regional perfusion (NRP). Direct procurement and preservation (DPP) followed by mounting the DCD heart onto an ex-situ perfusion machine takes more time than restarting the circulation in situ^8^. During warm ischemia, the heart is active and depletes its intracellular energy stores rapidly. It has been documented that the incidence of moderate or severe ISHLT primary graft dysfunction (PGD) in the DCD transplants can range from 15 to 41%^6^, likely related to the period of warm ischemia that occurs at the beginning of the agonal phase to the infusion of cold cardioplegia solution. Interestingly, this higher rate of PGD does not appear to affect patient or graft survival at 30 days or 1 year in the overall population. It was not known, however, if these findings extended to the ACHD population.

Wisniewski and colleagues recently analyzed 420 ACHD patients from the UNOS registry and found worse 90-day post-transplant survival in the DCD compared to DBD group, in the context of longer ischemic times and higher use of ex-situ perfusion devices in the DCD group. PGD rates were similar but rejection rates were higher in the DCD group. Landmark analysis for 90-day survivors showed no additional detriment in survival in the DCD group. The findings did not appear to be due to the DCD learning curve, as 2020–2022 transplants showed no differences in outcomes between DCD and DBD cohorts, but the 2023–2024 DCD transplants had worse survival than DBD.^9^

In this issue of JHLTO, Berg et al. expand on the available literature by evaluating 90-day and 3-year mortality in 726 ACHD transplant recipients, which included 61 DCD recipients, using the UNOS database.^10^ Graft failure (defined as death or retransplant) was higher in the DCD cohort using both multivariable analysis and propensity score matching methods. The increased hazard was most prominent in the early post-transplant period, with 2.5 fold higher 90-day hazard in the DCD group Higher GFR at transplant was protective, and ECMO at listing had deleterious effect (hazard ratio 3.92).

Understanding the mechanisms underlying these results remains a critical next step. Center volume has been previously shown to predict outcomes in ACHD patients. To adjust for regional ACHD experience, Berg et al followed the methodology of Nguyen et al^11^ to create a binary indicator of highest-volume ACHD center per region. Although 42.6% of transplants occurred at highest-volume ACHD centers compared with 29.0% of DBD (p = 0.027), after adjustment, DCD remained independently associated with worse 90-day and 3-year graft survival. These findings, along with Wisniewski’s findings that later periods were not associated with lower DCD mortality, suggest that center volume and DCD experience don’t fully mitigate the higher risk in these patients.

The unique vulnerabilities of ACHD recipients, including abnormal cardiac anatomy, multiple prior sternotomies, elevated pulmonary vascular resistance, and higher allosensitization combined with DCD-specific ischemia-reperfusion injury and longer operative times lead to multiple-hit biologic rationale for the observed excess early mortality. The study underscores the need for cautious recipient selection and refinement of preservation and reperfusion techniques including NRP, hypothermic oxygenated perfusion, and beating heart techniques.^12^, ^13^, ^14^ It is also important to recognize that ACHD patients have longer wait times and higher waitlist mortality. Since DBD donors are not always available, decisions must also weigh the potential hazards of DCD grafts against the ongoing mortality risk of waiting for the ideal organ.

A more granular and disease-specific approach is essential to fully understand the challenges facing ACHD patients. Current UNOS data, while invaluable, lack detailed perioperative, anatomical, and hemodynamic information that could illuminate the impact of specific congenital substrates—such as single-ventricle physiology, Fontan circulation, and multiorgan involvement—on outcomes. Future investigations must extend beyond registry-level analyses to capture these critical nuances and guide evidence-based strategies for this uniquely complex population.

This study highlights the challenges of combining a vulnerable DCD heart with a vulnerable ACHD patient, both with increased up-front risk and reduced reserve for hemodynamic and other insults.

DCD transplantation offers promise for expanding the donor pool, but in ACHD patients, the risks remain tangible; realizing this potential safely will require innovation, rigorous study, and careful recipient selection.

## Disclosures

None.

## Funding

None.

## Declaration of Competing Interest

The authors declare that they have no known competing financial interests or personal relationships that could have appeared to influence the work reported in this paper.
